# Knowledge-based computational models

**DOI:** 10.18632/oncotarget.2276

**Published:** 2014-07-29

**Authors:** Wim Verhaegh, Anja van de Stolpe

**Affiliations:** Precision & Decentralized Diagnostics, Philips Research, Eindhoven, The Netherlands

In our recent paper [1], we describe a biology-driven approach to translate cancer genomic data into clinically actionable information for personalized patient therapy selection. Despite the increasing knowledge on intracellular signal transduction pathways as drivers of tumor growth, and the corresponding development of a large number of “targeted drugs” to correct aberrant pathway behavior in cancer, it appears to be a tremendous challenge to select treatment protocols with a sustainable outcome for many patients. As the number of treatment options and their combinations increases, it becomes even more necessary to develop approaches for such “precision diagnostics”. Although extensive amounts of genomic microarray and sequencing data have been generated, which hold the key for optimal treatment selection for an individual patient, it appears to be difficult with presently available approaches to translate complex genomic data into clinically meaningful results.

Many genomic studies focus on identifying DNA mutations associated with therapy response and prognosis, however many results have failed to be clinically actionable [2]. First, most mutations found in tumors are passenger mutations, while only a few are driving tumor growth. Second, of only a limited number of tumor-driving mutations the incidence is sufficiently high to allow clinical validation, while most mutations are highly patient specific. Furthermore, the cancer genotype is increasingly recognized as providing only part of the puzzle. Other aspects such as the tumor micro-environment are thought to be equally important in determining functional behavior of cancer cells. As result, combined interpretation of cancer genotype and molecular phenotype is required to fully characterize an individual tumor and enable reliable prediction of therapy response [2].

In addition to the above, most efforts to identify diagnostic or predictive biomarkers are data driven, and fail to exploit the rapidly expanding biological cancer knowledge. We argue that this may be a costly omission, since using genomics data within a cancer biology knowledge framework enables reduction of data noise and focused extraction of relevant information for tumor characterization. Furthermore, the huge number of available data features versus the number of patient samples, combined with the fact that such sample sets are generally very heterogeneous, makes a data-driven approach prone to finding spurious patterns. Limiting biomarker search by using biological knowledge leads to more robust findings, which can be translated more straightforwardly into clinical practice as diagnostic assays.

In our paper [1], we address the primary question to be answered when predicting which targeted drug works best for a patient: which biological pathway(s) is/are deregulated and driving tumor growth? To this end, we have developed knowledge-based Bayesian computational models representing transcriptional programs of cellular signaling pathways, starting with well-validated direct pathway target genes. The expression levels of these target genes in a cancer tissue sample are a direct consequence of corresponding pathway activity, and their mRNA levels are used by the models to infer a probability of activity for each pathway. After calibration of the models on (very) limited numbers of training samples with established pathway status, we show that they can reliably assess signaling pathway activity in tumors of different tissue origin. The main difference between our approach and mainstream pathway analysis approaches is that we interpret mRNA data as expression levels of the pathways' target genes, while most mainstream approaches interpret mRNA levels as a surrogate measurement for levels of corresponding (active) signaling proteins, even though the correlation between (activated) protein and mRNA levels is known to be weak. Compared to data-driven approaches, the Bayesian network approach has the advantage of easier adaptation in case additional knowledge and/or data is to be incorporated. For instance, the models can be extended with additional parts of the signaling cascade to factor in effects of gene mutations, or tissue-specific target genes can be added.

Clinical validation of predictive and prognostic biomarker profiles is a difficult task, with uncertain factors such as population/tumor heterogeneity and long patient follow-up periods. Our approach, however, enables validation of the models in two stages: (i) biological validation of pathway activity assessment, followed by (ii) validation of clinical utility of the pathway test. Biological validation can be performed most effectively on well-defined cell line model systems allowing tight control of pathway status. This increases chances of successful clinical validation on patient samples in the second stage. In this way, we show that Bayesian models of the canonical Wnt and estrogen receptor (ER) pathway correctly assess pathway activity across various cancer tissue types and cell line experiments, after which we show that ER pathway activity is associated with better prognosis for ER-positive breast cancer patients treated with adjuvant hormonal therapy [1].

Our computational pathway models can be used in several ways. First, to predict response to pathway-targeted therapy, e.g. in neoadjuvant settings, and to monitor therapy response using a repeat biopsy after installing treatment, where in case of response a reduction in pathway activity is expected. Second, for prognosis and to measure potential therapy resistance, e.g. ER-positive breast cancer patients may not respond to hormonal therapy if another pathway like Wnt is active and driving tumor growth instead, and Wnt activity hence indicates a worse prognosis. The models may also be used to guide additional diagnostics, e.g. the search for tumor-driving mutations can be focused on the pathway(s) that show abnormal activity, reducing noise caused by passenger mutations in other pathways.

In summary, by interpreting genomics data in the context of the biology underlying cancer growth, we derive more clinically actionable information to select targeted therapies for individual patients, bringing “precision diagnostics” a step closer.
